# Pregestational BMI Over 23 kg/m2 May Increase Insulin Utilization in Gestational Diabetes Mellitus Patients

**DOI:** 10.7759/cureus.75612

**Published:** 2024-12-12

**Authors:** Risa Nakamura, Tsuyoshi Ohkura, Sonoko Kitao, Shoichiro Izawa, Takashi Harada, Shin-Ichi Taniguchi, Fuminori Taniguchi, Tasuku Harada, Kazuhiro Yamamoto

**Affiliations:** 1 Department of Endocrinology and Metabolism, Faculty of Medicine, Tottori University, Yonago, JPN; 2 Department of Obstetrics and Gynecology, Faculty of Medicine, Tottori University, Yonago, JPN; 3 Department of Community-Based Family Medicine, Faculty of Medicine, Tottori University, Yonago, JPN; 4 Department of Cardiovascular Medicine, Faculty of Medicine, Tottori University, Yonago, JPN

**Keywords:** homa-ir, insulin resistance, insulin treatment, postpartum glucose tolerance, pregestational bmi

## Abstract

The purpose of this study is to examine the pregestational BMI value that results in insulin use in Japanese patients with gestational diabetes mellitus (GDM) and to assess whether the type of GDM treatment affects postpartum glucose tolerance.

This retrospective study included 21 GDM patients treated until parturition at our department from 2013 to 2017. We calculated the pregestational BMI related to insulin treatment and the significant elevation in homeostasis model assessment of insulin resistance (HOMA-IR) by receiver operating characteristics curve (ROC) analysis. We also analyzed whether the insulin treatment caused a difference in postpartum glucose tolerance. Seven of the eight patients who needed insulin treatment had a pregestational BMI over 23 kg/m^2^. The pregestational BMI cutoff value related to insulin treatment was 22.5 kg/m^2^ (sensitivity 100%, specificity 46.2%, area under the curve 0.668, and Confidence Interval=0.429-0.907) in ROC analysis. Insulin utilization was significantly higher in the group with a pregestational BMI of 22.5 or more (p=0.045). HOMA-IR at postpartum was higher in patients whose pregestational BMI was 22.5kg/m^2^ or more. Blood glucose levels, HOMA-IR, homeostasis model assessment of β-cell function (HOMA-β), and the insulinogenic index (IGI) after delivery did not differ between the diet and insulin treatment groups. In conclusion, Japanese women with GDM and a pregestational BMI over 23 kg/m^2 ^may increase the risk of requiring insulin treatment during pregnancy. Postpartum glucose tolerance did not differ between patients treated with diet or insulin treatment for GDM.

## Introduction

Obesity is cited as a risk factor for gestational diabetes mellitus (GDM), and the increased risk is thought to be associated with insulin resistance in GDM. Many studies have reported that insulin treatment is required if the pregestational BMI is high [1]. Insulin resistance is defined as a state in which insulin action is reduced. Homeostasis model assessment of insulin resistance (HOMA-IR) is often used as an evaluation of insulin resistance in pregnant women. Recently, insulin resistance in GDM patients with an average BMI of about 25 kg/m^2^ has been reported in Canadian [2]. Moreover, in some reports of patients with GDM, many had a BMI of 25 kg/m^2^ or less [3].

According to a survey released by the Ministry of Health, Labour and Welfare in 2015, the majority (67.5%-77.8%) of Japanese women aged 20-49 years have a BMI of 18.5-24.9 kg/m^2^ [4]. In this regard, it has been reported that Asian individuals, including Japanese individuals, have increased insulin resistance at a lower BMI than Western individuals [5]. In 2015, the American Diabetes Association reported that Asian Americans have an increased risk of developing diabetes at a BMI of 23 kg/m^2^ or more [6]. Furthermore, insulin resistance increases at approximately a BMI of 23 kg/m^2^ in Japanese individuals with type 2 diabetes mellitus [7]. Japanese women with GDM and a BMI over 25 kg/m^2^ can experience increases in insulin resistance requiring insulin treatment. However, few reports have focused on this issue.

Pregnant women with abnormal glucose metabolism are required to maintain their blood glucose levels as close to normal as possible while avoiding hypoglycemia. Treatment for GDM involves diet therapy, but if blood glucose management proves difficult, insulin treatment is prescribed. GDM patients are at risk of developing diabetes after parturition, and more than 10% are said to have a diagnosis of impaired glucose tolerance (IGT) shortly after parturition. However, there are few reports on the association between insulin treatment during pregnancy and postpartum glucose tolerance. Therefore, in Japanese women with GDM, we examined the pregestational BMI value that results in insulin use and assessed whether the type of treatment affects postpartum glucose tolerance.

## Materials and methods

Patients

This study was a retrospective, observational study including 21 participants. Six participants had been diagnosed with GDM after a 75g-OGTT (oral glucose tolerance test) at a different hospital and were then referred to the Division of Obstetrics and Gynecology in our hospital. Women with multiple pregnancies, type 1 diabetes, or those who needed ritodrine hydrochloride were excluded. The Ethics Committee of the Faculty of Medicine of Tottori University approved the study (approval number 22A118), which we conducted in compliance with the ethical principles of the Declaration of Helsinki. We obtained informed consent from all participants using a procedure approved by the Ethics Committee.

We explain and consent to the study when a patient visits us for the first time in clinical practice. Height and body weight were checked, and interviews were undertaken to obtain information on pregestational body weight, and previous pregnancies. Blood samples were collected from patients who attended our department after being diagnosed with GDM at our hospital or another hospital. In our hospital, blood samples were collected before loading, after 30 minutes, after 60 minutes, and after 120 minutes in the 75g-OGTT. Glucose and serum immunoreactive insulin (IRI) were also measured at the same time. We measured IRI by Chemiluminescent enzyme immunoassay (human insulin chemiluminescent immunoassay kits; BECKMAN COULTER, Tokyo, Japan) to calculate HOMA-IR, homeostasis model assessment of β-cell function (HOMA-β). The blood sample was collected after overnight fasting, and weight was measured at the time of the first examination and before parturition. Since this study design was targeted at patients after diagnosis of GDM, the time of diagnosis varied depending on the case from early to late stages of pregnancy.

HOMA-IR was calculated as follows: fasting plasma glucose (FPG) (mmol/L)×fasting IRI (F-IRI) (pmol/L)/22.5. HOMA-βcell was calculated as follows: F-PI (mmol/L)×20/FPG (μU/mL)−3. HOMA-IR and HOMA-β were evaluated in pregnant women [3,8]. Lastly, the insulinogenic index (IGI) was measured as follows: (insulin (pmol/L) at 30 min-insulin (pmol/L) at 0 min)/(glucose (mmol/L) at 30 min-glucose (mmol/L) at 0 min). As mentioned previously, serum IRI was not measured in all cases in this study. HOMA-IR and HOMA-β were measured in 16 cases, in which serum IRI was measured only on a fasting stomach in the 75g-OGTT, and IGI was calculated in 11 cases, in which serum IRI was measured at 30 minutes after loading. BMI was calculated by dividing weight by the square of height in meters. The amount of change in BW (ΔBW) during pregnancy was also calculated. As a treatment, we prescribed a daily calorie intake of 30 kcal per ideal body weight at first, and to start self-measurement of blood glucose at fasting and two hours. If the glucose value exceeded 120 mg/dL two hours after eating, patients were guided to eat a divided meal, with 80 kcal of the meal distributed two hours after the meal [9]. If blood glucose levels continued to rise, patients were guided to use insulin.

Statistics

Statistical analysis was performed using Easy R (EZR) and GraphPad Prism (version 9.0, San Francisco, La Jolla California USA) [10]. Data are expressed as mean± standard deviation (SD) of the mean. A *p*-value of <0.05 was considered to be statistically significant in all analyses. A t-test was performed to compare the mean values between the two groups. The Fisher test was also used to assess whether insulin utilization was biased between the two groups of pregestational BMI. Receiver operating characteristics curve (ROC) analysis was performed to assess BMI cutoff values for increased insulin utilization.

## Results

The basal characteristics of the participants are shown in Table 1. The mean age of patients was 34.3±5.67 years and pregestational BMI was 24.7±4.39 kg/m^2^. The mean HbA1c of participants was 32.3±15.56 mmol/mol, and in cases in which the patient already had advanced anemia at the first visit, glycoalbumin was measured (six participants). IRI at fasting was measured in 16 cases at diagnosis. Maternity hospitals do not tend to measure serum insulin levels when performing 75g-OGTT. In this study, five patients were diagnosed at other hospitals, and fasting IRI was only measured in four. Therefore, parameters, such as HOMA-IR, could not be evaluated in all cases.

**Table 1:** Clinical characteristics of 21 GDM patients The values are presented as mean±SD. ΔBW represents the weight change from pre-pregnancy to delivery. Abnormal points indicate the number of measurements in which blood glucose levels exceeded the GDM diagnostic criteria in the 75g-OGTT. A p-value <0.05 indicates statistical significance. FPG: fasting plasma glucose; PG: plasma glucose; IRI: Immunoreactive Insulin; IGI: insulinogenic index; OGTT: oral glucose tolerance test; GDM: gestational diabetes mellitus

Variables	Values
Maternal age at pregnancy (years)	34.3±5.67
Pregestational BMI (kg/m^2^)	24.7±4.39
ΔBW (kg)	6.1±3.3
Family history/yes	5
Past delivery/yes	5
History of GDM/yes	1
Week of diagnosis	20.5±8.2
First trimester (n)	9
Second trimester (n)	4
Third trimester (n)	8
HbA1c (mmol/mol) (n=16)	32.3±15.56
GA (%) (n=6)	12.0±3.0
75g-OGTT at diagnosis	
FPG (mmol/L)	4.8±0.59
30min-PG (mmol/L)	8.6±0.96
60min-PG ( mmol/L)	9.7±1.84
120min-PG ( mmol/L)	9.5±1.95
Fasting IRI (pmol/L)	39.0±32.53
30min-IRI (pmol/L)	229.4±244.56
60min-IRI (pmol/L)	295.2±282.49
120min-IRI (pmol/L)	476.6±503.71
number of abnormal points	1.57±0.74
Abnormal point=1 (n)	12
Abnormal point=2 (n)	6
Abnormal point=3 (n)	3
HOMA-IR (n=16)	1.68±1.15
HOMA-β (n=16)	159.17±163.74
IGI (n=11)	0.66±0.33

First, we examined the patient background and parameters at the time of GDM diagnosis and glucose tolerance after delivery in the diet and the insulin treatment groups (Tables 2, 3). Although the insulin-treated group tended to have a higher pregestational BMI, there was no significant difference between the two groups. HbA1c was significantly higher in the insulin treatment group. The glucose level at one hour in the 75g-OGTT was significantly higher in the insulin treatment group than in the diet therapy group. Abnormal 75g-OGTT values were also significantly higher in the insulin treatment group than in the diet therapy group. Three of the four cases with one abnormal point at fasting with the 75g-OGTT could be managed with diet therapy only. The other patient with one abnormal point at fasting required insulin treatment, and the pregestational BMI of the participants was 25.6 kg/m^2^.

**Table 2:** Comparison of glucose tolerance at the time of diagnosis between the two treatment groups ^*^p-value <0.05 indicates statistical significance. ^a ^represents the t-test. ^b^ represents the Fisher test. ΔBW represents the weight change from pre-pregnancy to delivery. Abnormal points indicate the number of measurements in which blood glucose levels exceeded the GDM diagnostic criteria in the 75g-OGTT. GDM: gestational diabetes mellitus; FPG: fasting plasma glucose; PG: plasma glucose; HOMA-IR: homeostasis model assessment of insulin resistance; HOMA-β: homeostasis model assessment of β-cell function; HbA1c: glycated hemoglobin; OGTT: oral glucose tolerance test; NA: not applicable

	Diet	Insulin	P-value
n	13	8	NA
Age (years)	34±4	34±7	0.979^a^
History of GDM/yes	0	1	0.381^a^
HbA1c (mmol/mol) (n=15)	30.3±16.68	40.9±3.27	0.037*^a^
Pregestational BMI (kg/m^2^)	23.8±4.80	26.2±3.21	0.238^a^
Pregestational BMI ≥22.5 (kg/m^2^) (n)	7	8	0.045*^b^
ΔBW (kg)	6.7±3.58	5.0±2.53	0.210^a^
75g OGTT at diagnosis			
FPG (mmol/L)	4.8±0.58	4.7±0.64	0.652^a^
30 min PG (mmol/L)	8.6±0.88	8.7±1.15	0.862^a^
60 min PG (mmol/L)	8.6±1.90	10.8±0.93	0.010*^a^
120 min PG (mmol/L)	9.3±2.27	9.6±1.71	0.781^a^
HOMA-IR	1.48±1.04 (n=8)	2.0±1.21 (n=8)	0.249^a^
HOMA-β	111.78±72.37 (n=8)	215.43±212.08 (n=8)	0.177^a^
Number of abnormal values	1.3±0.6	2.0±0.8	0.035*^a^

**Table 3:** Comparison of glucose tolerance at the time of postpartum between the two treatment groups The data are presented as mean±SD. A t-test was performed. ΔBMI: change in BMI; IRI: immunoreactive insulin; FPG: fasting plasma glucose; PG: plasma glucose; HOMA-IR: homeostasis model assessment of insulin resistance; HOMA-β: homeostasis model assessment of β-cell function; IGI: insulinogenic index; NA: not applicable

	Diet	Insulin	P-value
N	12	5	
ΔBW (kg)	6.7±3.58	5.0±2.53	0.216
75g-OGTT at postpartum			
FPG (mmol/L)	4.8±0.3	5.1±0.8	0.352
30 min PG (mmol/L)	7.6±1.5	8.8±0.9	0.108
60min PG (mmol/L)	7.8±1.6	9.6±1.8	0.064
120 min PG (mmol/L)	6.6±1.2	8.0±1.3	0.061
Fasting IRI (pmol/L)	33.0±23.88	34.3±17.96	0.965
30 min IRI (pmol/L)	209.5±123.44	292.3±195.64	0.390
60 min IRI (pmol/L)	253.5±116.77	243.0±90.93	0.527
120 min IRI (pmol/L)	249.4±190.66	343.5±175.86	0.493
HOMA-IR	1.0±0.8	1.2 ±0.8	0.807
HOMA-β	66.2±44.1	71.1±42.9	0.835
IGI	1.4±2.8	0.5±0.3	0.481

**Figure 1:**
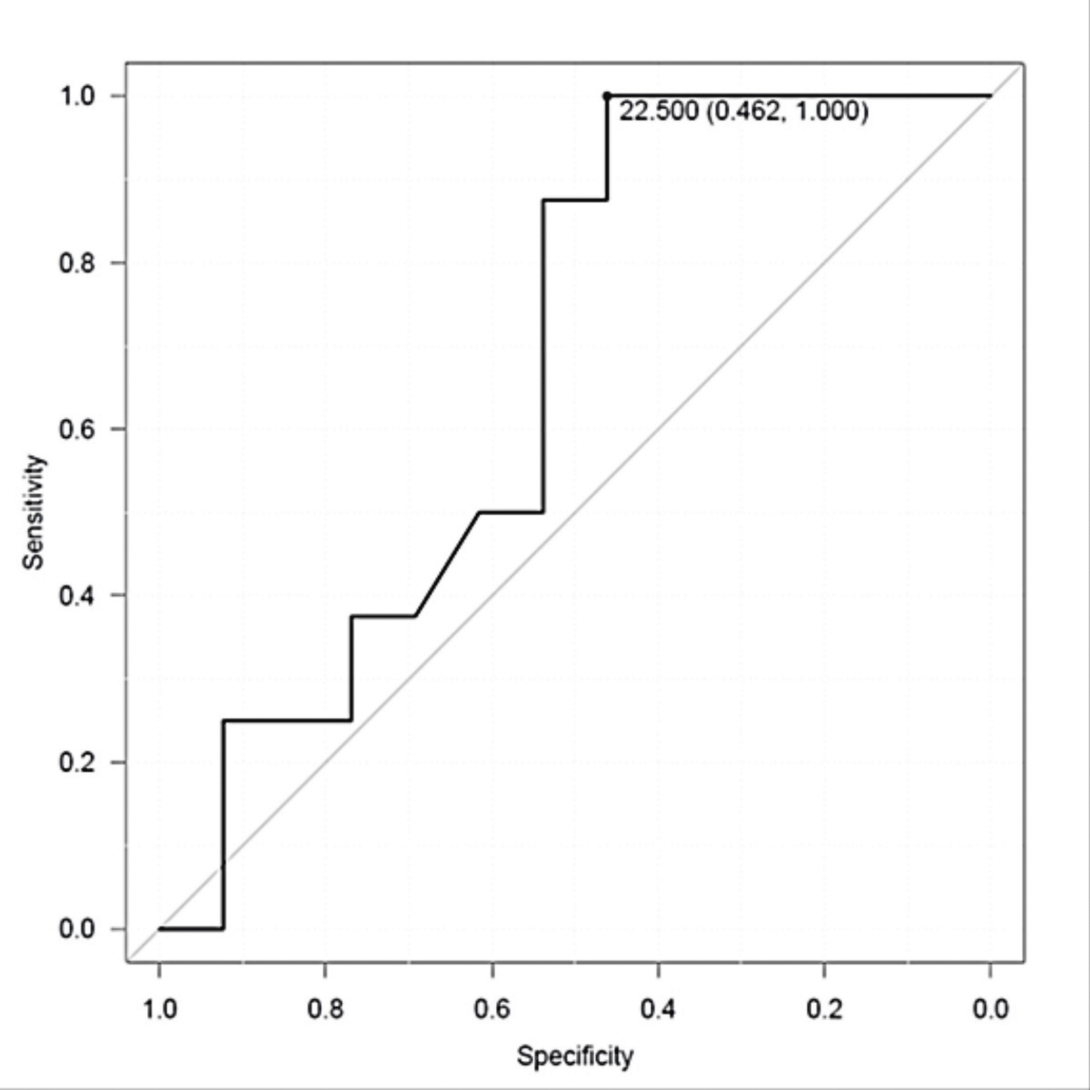
The cutoff level of the BMI related to insulin treatment in the ROC analysis ROC: receiver operating characteristics curve

All participants who needed insulin treatment had a BMI above 22 kg/m^2^. The BMI cutoff value related to insulin treatment was 22.5 kg/m^2^ (sensitivity 100%/specificity 46.2%, area under the curve (AUC)=0.668), 95% Confidence Interval (CI=0.429-0.907) in ROC analysis (Figure 1). Furthermore, the difference in insulin utilization was statistically significant when participants were divided into two groups based on a BMI of 22.5 kg/m², as determined by the Fisher test (*p*=0.048, the odds ratio=12.0). The mean postpartum HOMA-IR was also significantly higher in the group with a pregestational BMI of 22.5 kg/m^2^ or more. The mean HbA1c at diagnosis was significantly higher in the insulin-treated group (*p*=0.037). The cutoff value of HbA1c at diagnosis was 5.6 in ROC analysis (sensitivity 100 %/specificity 66.7%, AUC=0.875), 95% CI (CI=0.661- 1). There was no significant trend in insulin treatment related to the time of diagnosis. Blood glucose levels, serum IRI, HOMA-IR, HOMA-β, and IGI after delivery did not differ between the diet and insulin treatment groups.

## Discussion

In this study, the pregestational BMI cutoff value related to insulin treatment was 22.5 kg/m^2^ in ROC analysis. Insulin utilization was also higher in patients with a pregestational BMI of 22.5 kg/m^2 ^or more. The mean postpartum HOMA-IR was also significantly higher in the group with a pregestational BMI of 22.5 kg/m^2^ or more. Blood glucose levels, serum IRI, HOMA-IR, HOMA-β, and IGI after delivery did not differ between the diet and insulin treatment groups.

Although an association between obesity before pregnancy and the need for insulin treatment has been reported previously, it is unclear what cutoff value of pregestational BMI influences insulin utilization during pregnancy [1]. Interestingly, the World Health Organization has categorized overweight as a BMI of 25 or higher and obesity as a BMI of 30 or higher but tolerates lower cutoff values of overweight and obesity in Asia. Asians tend to have higher body fat than Caucasians even with the same BMI, and a cutoff value of overweight in Asian individuals of BMI 22 kg/m^2^ to 23 kg/m^2^ is more appropriate than over 25 kg/m^2^ [11]. This difference is confirmed by examining the threshold for increased cardiovascular risk. In fact, the prevalence of obesity-related diseases such as type 2 diabetes mellitus is high in Japan even though there are fewer people with high levels of obesity than in Europe or the United States. Consequently, it is considered appropriate to diagnose obesity with stricter criteria.

The average BMI of Americans with diabetes enrolled in the Third National Health and Nutrition Examination Survey was 26 kg/m^2^ and of British individuals with diabetes in the United Kingdom Prospective Diabetes Study was 27.5 kg/m^2^ [12,13]. By contrast, according to Japan Diabetes Data Management, the average BMI of Japanese individuals with diabetes in 2013 was 25.0 kg/m^2^. There are many GDM patients with a pregestational BMI of 25 kg/m^2^ or less in Japan. However, data are lacking on whether that degree of pregestational BMI is related to insulin resistance or the need for insulin treatment after pregnancy. Therefore, future discussions are necessary.

As the indicator of insulin resistance, the euglycemic hyperinsulinemic clamp method is the gold standard for evaluating tissue-specific insulin sensitivity [14]. However, the clamp method is very cumbersome and not recommended for pregnant patients. HOMA-IR is easily measurable and an indicator of insulin resistance, so this index was used. HOMA-IR is the most commonly used indicator of insulin sensitivity, mainly in the liver.

In a previous report, pregestational BMI was strongly associated with HOMA-IR in pregnancy in GDM patients [15]. However, patients were divided into two groups with a pregestational BMI of 25 kg/m^2^ as a cutoff, as in many other reports. Whether similar associations exist in patients with lower BMIs needs to be evaluated. In this study, the BMI cutoff value related to insulin treatment was 22.5 kg/m^2^ in the ROC analysis. Insulin utilization was higher in patients with a pregestational BMI of 22.5kg/m^2^ or more. Furthermore, HOMA-IR at postpartum was higher in patients whose pregestational BMI was 22.5 kg/m^2^ or more. From these results, we suggest that a pregestational BMI over 23 kg/m^2^ poses a risk in terms of insulin resistance in GDM patients. In the group with a BMI of 22.5 or higher, postpartum HOMA-IR was significantly higher (pregestational BMI <22.5 kg/m^2^: 0.46±0.29, pregestational BMI ≥22.5 kg/m^2^: 1.33±0.74, p=0.023). Furthermore, pregestational BMI was positively correlated with postpartum HOMA-R (p=0.67). There are few reports on the association between prenatal obesity and HOMA-IR within 12 weeks postpartum. However, HOMA-IR≥1.6 in 75g-OGTT at 12 weeks postpartum was considered to be at risk of developing type 2 diabetes within two years of follow-up [16]. In the Asian region, especially in developing countries, it is preferable to estimate the risk of pregnancy using simple indicators, rather than costly tests. In this respect, pregestational BMI may be related to insulin utilization during pregnancy and is important in practice. In this study, patients were followed up to 12 weeks postpartum. Consequently, studies with long-term review are necessary. Some large-scale studies targeted GDM have followed up until postpartum OGTT results, while little is known about the difference in glucose tolerance by treatment [17]. In this study, we reported that postpartum glucose tolerance does not differ depending on the presence or absence of insulin treatment. However, it is necessary to examine more cases.

The mean HbA1c at diagnosis was significantly higher in the insulin-treated group. Some reports have reported that HbA1c at diagnosis was significantly higher in the insulin use group [18]. This study included patients who had advanced gestation age at diagnosis and already had anemia. In those cases, GA was measured instead of HbA1c. Because the number of cases in which HbA1c was measured was limited, a sufficient analysis could not be conducted.

The study had some limitations. First, our facility is a university hospital, and only patients at increased perinatal risk are referred to the department. Therefore, the number of patients participating in the study was limited. In addition, cases of imminent premature birth after participation were included in the 21 participants. Furthermore, the timing of referrals to our department varied. Some referrals were made after the period when insulin resistance increases in the second trimester, making it difficult to evaluate insulin resistance at the time of the initial diagnosis. Many studies that control the intervention timing have considered the relationship between BMI in early pregnancy and GDM incidence in the second trimester of pregnancy. However, weight loss may occur in early pregnancy because of morning sickness. By contrast, we considered the effects of BMI just before pregnancy on IGT after pregnancy.

In addition, in the case of referrals from other facilities, serum IRI was not measured with 75g-OGTT at the time of diagnosis. Therefore, the number of patients whose HOMA-IR, HOMA-β, and IGI had been measured was also limited. In the future, to accurately evaluate insulin resistance and insulin sensitivity, the number of participants studied should be increased and the intervention timing should be unified.

## Conclusions

Japanese women with GDM and a pregestational BMI over 23 kg/m^2^ had an increased risk of insulin treatment for GDM. Insulin resistance increases at approximately a BMI of 23 kg/m^2^ in Japanese individuals with type 2 diabetes mellitus. An important point to note is that the risk of needing insulin treatment may rise in the case of pregestational BMI over 23 kg/m², even in nondiabetic patients. Postpartum glucose tolerance did not differ between the diet and insulin treatment groups.
